# Operations research meets need related planning: Approaches for locating general practitioners’ practices

**DOI:** 10.1371/journal.pone.0208003

**Published:** 2019-01-09

**Authors:** Melanie Reuter-Oppermann, Stefan Nickel, Jost Steinhäuser

**Affiliations:** 1 Karlsruhe Service Research Institute (KSRI), Karlsruhe Institute of Technology (KIT), Karlsruhe, Germany; 2 Institute of Family Medicine, University Hospital Schleswig-Holstein, Campus Lübeck, Germany; University of Botswana Faculty of Medicine, BOTSWANA

## Abstract

**Background:**

In most western countries a shortage of general practitioners (GP) exists. Newly qualified GPs often prefer to work in teams rather than in single-handed practices. Therefore, new practices offering these kinds of working conditions will be attractive in the future. From a health care point of view, the location planning of new practices will be a crucial aspect. In this work we studied solutions for locating GP practices in a defined administrative district under different objectives.

**Methods:**

Using operations research (OR), a research discipline that originated from logistics, different possible locations of GP practices were identified for the considered district. Models were developed under two main basic requirements: that one practice can be reached by as many inhabitants as possible and to cut down the driving time for every district’s inhabitant to the next practice location to less than 15 minutes. Input data included the demand (population), driving times and the current GP locations.

**Results:**

Three different models were analysed ranging from one single practice solution to five different practices. The whole administrative district can reach the central community “A” in at most 23 minutes by car. Considering a maximum driving time of 15 minutes, locations in four different cities in the district would be sufficient.

**Conclusion:**

Operations research methods can be used to determine locations for (new) GP practices. Depending on the concrete problem different models and approaches lead to varying solutions. These results must be discussed with GPs, mayors and patients to find robust locations regarding future developments.

## Introduction

Most western countries face a shortage of general practitioners (GPs) especially in rural areas [1, 2]. Strategies to provide sufficient, close to home care in the future to meet the needs of an aging society are needed [1, 3]. GPs in Germany can choose the location of their practice freely, if the district has not defined any restrictions. These restrictions are due to the ratio of GPs and inhabitants [4]. Newly qualified GPs in Germany tend to choose to work in a team rather than in single-handed practices and are more interested in an employment than having their own practice [5]. Especially female doctors request part-time employments. With 70% of all GPs being female doctors they will be the majority of future GPs [6]. However, in the past many GP practices in Germany were single-handed. As 30% of the GPs in Germany are older than 60 years, there are many single-handed practices that will not find a successor. For example in Baden-Württemberg, a federal state located in south-west Germany, around 34% of all GPs will retire in the next ten years. These approximately 2000 GPs are therefore looking for a successor for their practices as well as their patients. Worst case scenarios suggest that 500-1000 practices might be closed as there are not enough successors, leaving 750.000-1.500.000 patients with the need to find a new GP.

Primary health care in Germany is widely provided by GPs. Every inhabitant can freely choose their GP and about 90% do so [7, 8]. Unfortunately, the GP specialty is suffering from low applications, e.g. in a survey from 2014 only 8.9% of the medical students were planning on becoming a GP [9]. While different programs try to increase the attractiveness of the GP profession [10], it is also important to use the resources efficiently. Due to the demographic change even more GPs will be needed in the future to provide close to home primary medical care for the German population. Especially elderly patients with often multiple diseases are depending on receiving medical care by GPs and their number is constantly growing. While in 2000, 16.44% of the German population was 65 years or older, in 2010 it was already 20.63% [11]. Therefore, the Association of Federal State Health Authorities (AOLG) expects an increase of primary care demand of at least 20% in 2020 compared to 2000 [12]. The situation is even more critical in rural areas.

If GPs are a scarce resource, keeping or building practices for new GPs at strategically important locations is a necessity for efficient use of the resource. Mathematical approaches can help determining these locations. In this work we study the problem of locating GP practices in an administrative district in south-west Germany under different objectives using OR models. We present adequate mathematical models and use a realistic instance to demonstrate different aspects of location planning. Note that the results themselves do not aim to make any explicit suggestions for the considered region, as the focus of this work is on the location problem and the corresponding models.

**Fig 1:**
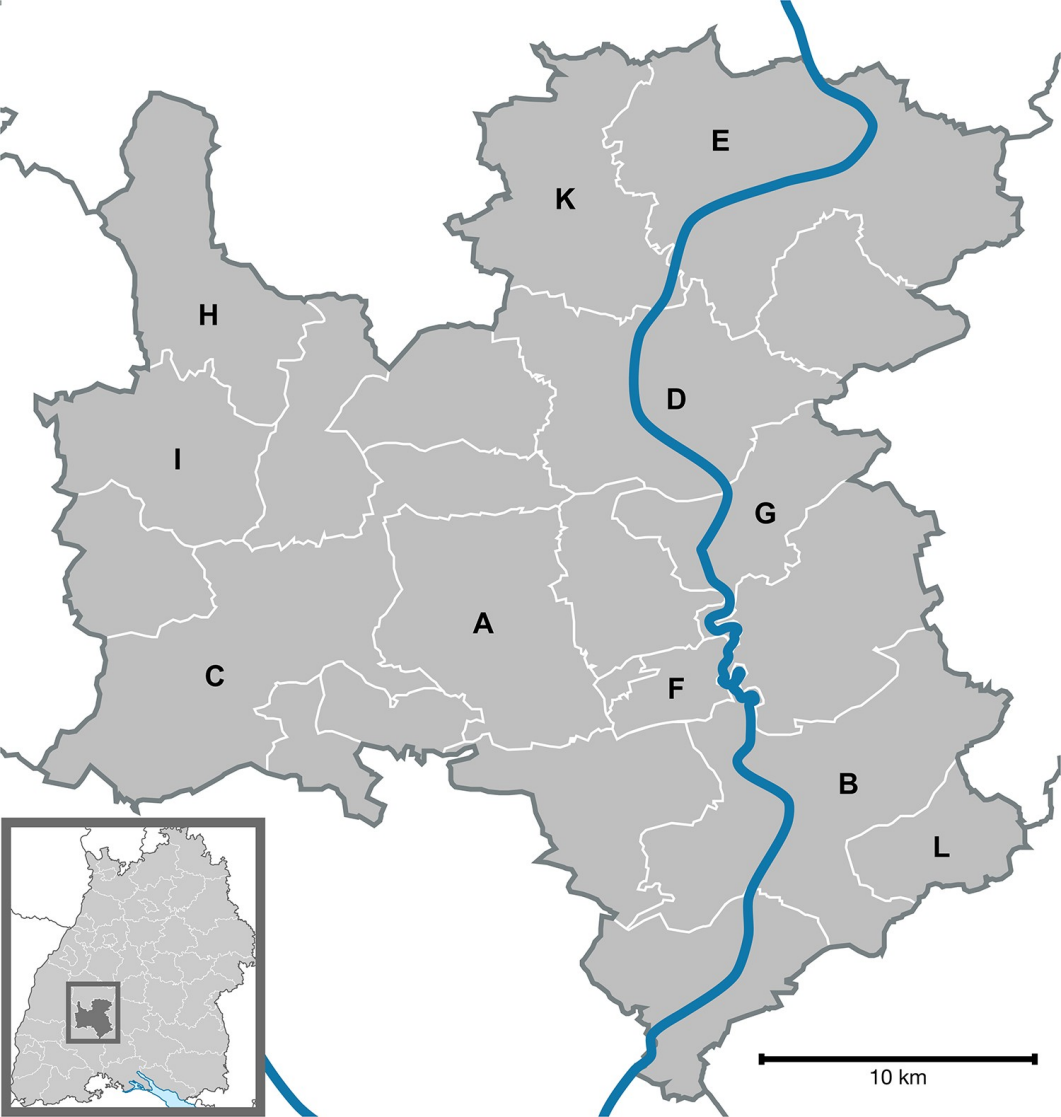
The administrative district and its 21 municipalities.

The administrative district under consideration in this work lies in the south of the previously mentioned federal state of Baden-Württemberg. Currently, 135,447 inhabitants are living in more than 500 different cities, villages and homesteads in this district. These are grouped into 21 small municipalities. Fig 1 shows the administrative district and its municipalities. The main two cities are located in “B” and “C”, additionally “D” and “E” are also important towns for providing basic services [13]. In addition, we labelled these municipalities with “A” and “F” to “L” that are included in at least one solution in the results section. The geographic conditions are embossed by the black wood forest with its mountains and valley on the one hand and the Swabian Alb with its low mountain range type of countryside.

This research is in line with a set of research questions addressed in the studied administrative district. First steps included a study on the demographical development within the district [14]. This was accompanied by a qualitative study aiming at collecting determinants for or against less common types of primary care practices in Germany, e.g. a practice model including more than three GPs and additional health care staff as employees [15]. Considering the GPs that want to work self-employed, about 90% of the GPs buy the practice from their older colleague. However, for modern primary care practices there is often a need to find a new location within the allowed region, for example when the existing practices are too small or too old or if the allowed locations are not convenient for such a new type of practice. Therefore, well-grounded logistic methods are needed to determine new locations including the necessary and sufficient number of locations for a region.

Determining locations for facilities is a well-studied problem in the area of operations research. In healthcare, models and approaches have been successfully applied to healthcare facility location problems, ambulance planning and hospital layout planning [16]. Based on [16], the objectives for locating healthcare facilities are

1. minimise the access cost for patients (e.g. travel cost, distance or travel time),
2. maximise covered demand, and
3. maximise equity in access.

## Methods and data

### Methods

We use operations research (OR) methods to determine future locations for general practitioners’ practices. Operations research is a discipline applying quantitative techniques in order to make the best possible decisions. Its origins lay in logistics using mathematical approaches [17]. It expresses a problem as a mathematical model usually including one objective function that is to be maximised or minimised and a set of constraints that need to be fulfilled. The model can then be solved by an open-source or commercial solver that determines the optimal solution for the problem, that is the best possible solution with respect to the objective.

In order to model the location problem we first need to express the studied region as a network *G* that contains a set of nodes *V* and a set of edges *E*. The nodes represent the 21 municipalities in the region and are set as the centers. The edges are weighted with the driving distances between the nodes. By *I* we denote the set of demand locations and by *J* the set of possible locations for a practice. In our case all nodes are demand locations as well as possible locations for practices and therefore *V* = *I* = *J*. Nevertheless, we will keep the notations of *I* and *J* for a better understanding. Each demand location *i* ∈ *I* has a weight *e*_*i*_ that must or should be fulfilled, depending on the concrete problem definition. In this work the demand at a node equals the population in the corresponding city or area. We say that it is covered if a located facility can be reached within a maximum time *r*_*max*_. It does not necessarily mean that patients are assigned to the located facility, only that they are potential patients for that practice. This is important as patients in Germany can choose their GP freely and are not assigned to a practice. If needed, the assignment of patients to practices or GPs can be easily included in the formulation. The driving time between a demand location *i* and a possible facility location *j* is denoted by *r*_*ij*_. This means that a facility located at *j* covers a demand node *i*, if *r*_*ij*_ ≤ *r*_*max*_.

We can determine one or several locations such that

1. the driving time for all patients is minimised,
2. the demand covered is maximised, or
3. the maximum time patients need to drive is minimised.

The three objectives can all be reasonably applied to the problem of locating GP practices.

The Maximum Coverage Location Problem (MCLP) by Church and ReVelle [18] locates *n* facilities in such a way that as much demand is covered as possible. In includes two sets of decision variables:

$$\begin{array}{c} x_{j}=\{\begin{array}{cc} 1, & \text{if}\;\text{a}\;\text{facility}\;\text{is}\;\text{located}\;\text{at}\;j\text{,}\\ 0, & \text{otherwise.} \end{array}\;\left(j\in J\right) \end{array}$$

$$\begin{array}{c} y_{i}=\{\begin{array}{cc} 1, & \text{if}\;\text{the}\;\text{demand}\;\text{location}\;i\;\text{is}\;\text{covered,}\\ 0, & \text{otherwise.} \end{array}\;\left(j\in J\right) \end{array}$$

It additionally needs the definition of a set *N*_*i*_ = {*j*|*r*_*ij*_ ≤ *r*_*max*_} that includes all possible facility locations *j* that can cover a demand node *i*.

The model then looks as follows:

$$\begin{array}{ll} \text{maximise} & \sum_{i\in I}e_{i}y_{i}, \end{array}$$ (1)

$$\begin{array}{ll} \text{subject}\;\text{to} & y_{i}\leq \sum_{j\in N_{i}}x_{j},i\in I, \end{array}$$ (2)

$$\begin{array}{cccc} & & & \;\sum_{j\in J}x_{j}=n, \end{array}$$ (3)

$$\begin{array}{ll} x_{j}\in \left\{0,1\right\}, & j\in J, \end{array}$$ (4)

$$\begin{array}{ll} y_{i}\in \left\{0,1\right\}, & i\in I. \end{array}$$ (5)

The objective function (1) maximises the covered demand. Constraints (2) assure that a demand location can only be covered if a suitable facility is located. *n* facilities must be located as stated by constraint (3). (4) and (5) are the domain constraints for the decision variables.

The MCLP gives us the locations for *n* GP practices and the number of municipalities covered assuming a maximum driving time *r*_*max*_. Note that by a covered municipality we only mean that all inhabitants can reach the new practice within the maximum driving time (and are therefore potential patients).

For the case study we also want to know the closest (new) practice for each municipality. Therefore, additional decision variables are needed that allocate demand nodes to located facilities:

$$\begin{array}{c} z_{ij}=\{\begin{array}{cc} 1, & \text{if}\;\text{the}\;\text{demand}\;\text{location}\;i\;\text{is}\;\text{assigned}\;\text{to}\;\text{facility}\;\text{location}\;j\text{,}\\ 0, & \text{otherwise.} \end{array}\;\left(i\in I,j\in J\right) \end{array}$$

In a second model we additionally added integer decision variables *a*_*j*_ that give the number of GPs needed at locations *j* ∈ *J*. Of course, the decision variable is 0 for all those locations where no practice is opened. Furthermore, we have a parameter *p* that denotes the number of patients that one GP can serve. For this model we assume a complete restructuring of the region and do not take current practices into account. To do so we explicitly need the allocation of patients to GPs and say that a demand point is always assigned to the closest facility, as the majority of patients (71.5%) consult the closest GP [19]. We assume that the patients that choose a different GP more or less equal out over the district. The model also weights the distances that the patients need to travel by the number of patients. Note that for this research question we assumed that all patients must be covered.

In a third formulation we also included the existing locations and modeled the time horizon as a changing number of inhabitants and GPs until 2023. We studied where new practices should be located (or kept open if already existing) and how many GPs are needed. We assumed that now, in 2017, 2020 and 2023 always one location can be chosen.

Note that with these models we focus on finding solutions that maximise the coverage and/or minimise the patients’ driving times. Maximising other aspects regarding the “attractiveness” from a patient’s point of view, e.g. existing infrastructure or proximity to their workplaces, could also be included into the model. In addition, it is also possible to include the doctor’s point of view into the models, e.g. considering the existing infrastructure that is of relevance to them. Further investigations are needed to determine relevant aspects for patients and doctors to be included into a location model. Therefore, the model extensions will be studied in future research.

The models were implemented and solved to optimality with the IBM CPLEX Optimization Studio.

### Data

The data was derived for the 21 municipalities in the district. The main input for the demand side were the numbers of inhabitants, presented in [14]. We had the concrete numbers for 2014 as well as the expected numbers for 2023 and calculated the values in between for three-year intervals. For *n*, values from one to four are used in this work. By allowing larger (or forcing smaller) maximum driving times, additional instances were created.

For the parameters *r*_*ij*_ the driving times (car) between the town halls were determined via the Google Distance Matrix API [20] and stored in a resulting distance matrix. The town halls were chosen as they are (usually) centrally located within a municipality. Note that in practice there can be a difference between distances and ride times, especially when different means of transport can be used. The use of public transport for example usually decreases with increasing distance of villages within rural areas. A pretest with patients in the federal state of Baden-Württemberg showed that 75% of the patients drive to their GP in their car. The majority of the remaining 25% walks. We chose 15 minutes as the reference value for the maximum driving time *r*_*max*_ as this was determined in a survey with mayors from that federal state [21].

For models 2 and 3 we additionally needed information about the current and future GPs. In general we assume that a GP can serve 1600 patients as the need related planning by the Association of Statutory Health Insurance Physicians uses this number [14]. We determined the current numbers of full-time GPs per municipality using the GP registry [22]. In 2014 60 GP practices existed in the district. In addition, we did telephone interviews with the existing practices to ask for the following information:

1. Only one general practitioner or collaborative practice,
2. number of general practitioners,
3. number of employed doctors,
4. number and age of GPs working full-time, and
5. number, degree of part-time [in %] and age of the GPs working part-time.

We needed this information in order to determine the expected year of retirement of the GPs assuming that the average age for retirement is 65. Another output was that we could adjust the current number of GPs.

## Results

It was possible to determine optimal solutions for the problem by solving the models with the described input data. Note that the solutions only display the functionality and the differences of the models. They should not be used as suggestions for the actual studied district.

### First model

The whole administrative district can reach “A” in at most 23 minutes by car while the average driving time is 14 minutes. There is no other location in the district with a shorter maximum driving time. Therefore, if only one health center should be built to be as close as possible for all patients, the location should be “A”. If 23 minutes are too much, more than one location is needed.

One can easily think of two alternative solutions for the single location problem. These locations maximise the covered percentage of the population within a given radius, i.e. a maximum driving time. For a maximum driving time of up to six minutes, “B” would be chosen as the single location due to its size. Taking the 15 minute reference value, the optimal location would be in “F” covering already 70% of the overall population. In this case the average driving time would be 10.5 minutes.

If two new locations can be built 90% of all inhabitants could be reached from “D” and “F” in 15 minutes. For a maximum of 18 minutes the whole districts could be reached and locations would be “C” and “G” with an average driving time of 9.6 minutes.

Another question that we studied was how many locations are necessary so that all patients can reach a new practice in at most 15 minutes. The result is that four new practices are necessary in “D”, “H”, “C” and “F” with an average driving time of 6.5 minutes. Allowing five or six new locations, the average driving times can be reduced to 6.4 or 5.7, respectively.

Table 1 gives an overview of the results for the first model.

**Table 1:** Overview of the results for the first model.

# locations	Max ride time	∅ ride time	% covered	Villages
1	6	–	–	“B”
1	15	10.5	70%	“F”
1	23	14	100%	“A”
2	15	–	90%	“D”, “F”
2	18	9.6	100%	“C”, “G”
3	18	–	100%	“C”, “D”, “F”
4	15	6.5	100%	“D”, “H”, “C”, “F”

### Second model

With one central practice in “A”, 87 GPs would be necessary to be able to treat all patients. In the case of the two locations “C” and “G”, 30 and 57 GPs would have to be employed. When four new locations are requested these would be located in “D” (27 GPs), “B” (32 GPs), “I” (4 GPs) and “C” (25 GPs), i.e. overall 88 GPs would be located. The maximum ride time would be 15 minutes, the average being 5.3 minutes. If we allow 21 locations, the number of GPs needed would increase to about 96, meaning of course that not all GPs would have a full patient panel of 1600 patients. With five locations, only 89 GPs would be necessary. Considering the average ride times, ten locations would lead to 1.93 minutes and only five locations to 4.2 minutes. One can say that from a ride time point of view, five locations for practices in the district would be fine.

The results show that for the second part of the study the locations “B”, “C” and “D” are most important.

### Third model

Now we take the future developments (inhabitants and GPs) into account. If we allow all possible locations in the district, we see that mostly very small municipalities are chosen as they explicitly lack GPs. Assuming 2014 as the status quo, six new GPs should have started in “K” by 2017. In 2020, ten GPs should be located in “G” and by 2023 five additional GPs should work in “E”. In 2023 a practice with two general practitioners should open in “L”.

In a second run we restricted the set of possible locations to the main cities “D”, “B”, “C” and “E” as they should be more attractive for the young general practitioners to settle as well as for the majority of patients as these cities already offer many other (healthcare related) services. By 2017, six new GPs would have to start in “E”, seven GPs in “B” by 2020 and by 2023 another seven again in “E”. In 2023 two additional GPs are required for “B”.

## Discussion and limitations

This research presents a study on locating GP practices using operations research models in a German district. It shows the applicability of OR to the planning problem. The assumption that patients choose the closest GP is valid for this work as the main focus was on the locations and not on the number of doctors needed or the explicit form of practice organisations. Nevertheless, it is of interest to include the patients’ behavior for choosing the GP into future models. For this district it was sufficient to take the driving times by car as a pilot-study showed that 3/4 of the patients drive to the GP. This number was also determined in a study for a different district in the same federal state [23]. The other 25% are those patients in our model that live within the city where a practice is located. For future studies with other districts and cities this might not be the case. Therefore, more research is needed on (1) how patients are travelling to the GP—maybe depending on the practice location and (2) how different means of transport can reasonably be included into the mathematical models. The study also shows that the results differ significantly even for this comparably small district, depending on the maximum driving time and on the number of new practices. We assumed that costs for opening a new practice are equal for all locations and we were looking for solutions from the patients’ point of view. While this was a realistic assumption for the district, it might not always be true. Sometimes already existing buildings (maybe even practices) might be used, in other cases the prices for real estate could vary between the villages or districts. Therefore, it might be interesting to build and test corresponding models that also take different cost structures into account.

While we exemplify possible results for a German district in this paper, the approaches can be transferred to basically every German district as well as to many other countries worldwide in which patients can choose their GP, for example to countries as far away as New Zealand. On the other hand, OR approaches can also be applied to countries in which patients are assigned to their GP, e.g., as in Turkey or Portugal. In some aspects the planning might actually be easier in these countries as no assumptions are necessary about how far patients are willing to travel to their GP and which factors besides the distance influence the choice of a GP.

For the first model the decrease of the average driving time is not significant, hence it does not seem reasonable to open more than four new practices. Looking at the set of solutions it becomes clear that the cities “C” and “F” are often chosen and are therefore important locations for the district. Concluding, if the aim is to now build one location and another one next year, for example, a first practice should not be built in “A”, but in “C” or “F” (and a second one in the other village, respectively).

Obviously, the second model is more of theoretical than practical value. Nevertheless, it gives an idea of the number of GPs needed for different scenarios without incorporating additional strategies such as shifting tasks from GPs to nurses, for example. Including the weighted traveled distances into the model can lead to different results for the chosen locations. In that way, the second model could also be considered to be relevant for the location decision itself neglecting the determined number of GPs.

With regard to the third model we expect that by 2023 many new GPs are needed to assure primary care coverage for all inhabitants.

The results of these models were discussed with the representatives of the local government (e.g. mayors). During the discussion it became clear that planning of practices includes also political aspects that need to be considered. Therefore, a way of moderating theoretical models into real practices is necessary, as stated by [3] and presented in [24].

The results show that it is crucial to be sure about the assumptions, constraints and objective(s)—for example regarding the demand or the time horizon—as the solutions can differ significantly. A location that might be optimal for one formulation might not even be included in the solution of another only slightly different formulation. Therefore, it is important to formulate the problem precisely and scrutinise the solutions, also regarding the assumptions and future developments.

## Conclusion

Operations research methods can reasonably be used to solve the problem of locating (new) GP practices. They can also help to decide how many GPs are needed to cover a region. The presented approaches can easily be transferred and adapted to the problem of locating GP practices in other regions and countries worldwide. Depending on the concrete problem different models and approaches might be needed that can lead to varying solutions. Therefore, it is important to specify the constraints and objective precisely and if possible, to also take future developments into account, if these can be quantified.
